# Homeostatic Plasticity in the Nervous System

**DOI:** 10.1155/2012/913472

**Published:** 2012-07-11

**Authors:** Arianna Maffei, Dirk Bucher, Alfredo Fontanini

**Affiliations:** ^1^Department of Neurobiology and Behavior, SUNY-Stony Brook, Stony Brook, NY 11794-5230, USA; ^2^Graduate Program in Neuroscience, SUNY-Stony Brook, Stony Brook, NY 11794, USA; ^3^State University of New York Eye Institute, Syracuse, NY 13202, USA; ^4^The Whitney Laboratory for Marine Bioscience, University of Florida, St. Augustine, FL 32080, USA; ^5^Department of Neuroscience, University of Florida, Gainesville, FL 32611, USA

Every organism relies on the maintenance of stable internal states, a phenomenon usually referred to as homeostasis. While homeostatic control of physiological functions like ion regulation has long been well described, homeostatic control of neuronal and network function is a relatively recent concept. It is intuitive to think that regulatory mechanisms must exist to preserve neural function in the face of constantly changing internal states and/or interactions with the environment. Growth and development, experience-dependent plasticity, and constant molecular turnover pose a challenge to the nervous system to keep all parameters within a functional range without overconstraining plastic processes vital for adaptive changes. Investigating the complexity of homeostatic regulation of single neurons and neural circuits is thus fundamental for understanding brain function. 

In recent years, there has been a dramatic increase in the number of contributions to the study of mechanisms underlying homeostatic plasticity in the brain. One of the remaining challenges is to integrate findings from different levels of analysis, possibly providing a comprehensive theory that encompasses the complexity and multitude of findings, which at times may appear contradictory. Multiple levels of homeostatic regulation have now been identified in a variety of model systems. Neurons can preserve their excitability in a functional dynamic range by adjusting their intrinsic properties and the strength of their synapses in a cell-autonomous manner. A number of cellular and molecular mechanisms have been identified as regulators of cell-autonomous homeostatic plasticity. In most systems, the activation of these mechanisms occurs after a sensor detects deviations from expected levels of electrical activity and begins a cascade of events that compensates for these changes. Systems in which the expected set-point activity of a single neuron has been directly correlated with network function have allowed the identification of a number of parameters that can be adjusted to maintain stable levels of activity in the nervous system. Perhaps not surprising for such a fundamental property of biological systems, there is no unique pathway to homeostatic regulation of network activity even in relatively simple circuits. When looking at different neuronal types that make up neural circuits, it is often found that cell-autonomous mechanisms for homeostasis differ substantially. For example, chronic manipulations of activity result in changes in the excitability and synaptic properties of glutamatergic cortical neurons, consistent with a cell-autonomous regulation towards a set-point. In contrast, the same manipulations result in changes of GABAergic neuron properties that do not seem to promote maintenance of their own excitability. However, these changes may effectively favor maintenance of network activity by appropriately adjusting the global balance of excitation and inhibition in the circuit.

In this issue, G. Wang et al. review the current literature regarding homeostatic synaptic plasticity and the mechanisms regulating AMPA receptors trafficking and discuss these findings in the context of homeostatic regulation of the excitability of excitatory neurons. T. E. Krahe et al. report a role for CREB in homeostatic synaptic plasticity and propose that this set of plasticity mechanisms in the dorsal lateral geniculate nucleus plays a major role in regulating the sensitivity of cortical circuits to changes in sensory drive. F. C. Roth and A. Draguhn highlight the importance of the regulation of GABA metabolism and transport for circuit homeostasis, and propose that inhibitory synapses may play a fundamental role that goes well beyond providing a brake on neural excitability.

The existence of multiple mechanisms for homeostatic plasticity is consistent with the possibility that each cell type may have an entire toolkit at its disposal to maintain a balanced level of activity, and it suggests a significant degree of flexibility in how a network can respond to different challenges. This diversity of mechanisms makes it difficult to identify general rules for homeostatic plasticity. Therefore, the investigation of interactions between different neuron types and their role in network function is crucial. Although the possibility that there are fundamental constraints to the degree of variability and of coordination of different homeostatic changes has been explored, this area of research is still in its infancy. Here, theoretical work is of fundamental importance for the integration of diverse experimental findings, with the goal to provide a general conceptual framework for homeostatic regulation of circuit excitability and function. In this context, B. N. Queenan et al. employ the principles of control theory to formally describe basic features of circuit homeostasis in the attempt to provide a comprehensive theory.

At the systems level, the interaction between cell-autonomous and circuit mechanisms may preserve stable sensory, motor, and cognitive functions. Investigating how homeostatic mechanisms observed at the single neuron and circuit level are integrated to regulate brain activity is extremely challenging. The complexity of interactions between different brain areas in sensory and cognitive processing and the difficulty of relating synaptic and intrinsic forms of plasticity to complex network functions have limited our ability to bridge cellular and system levels. Brainwide synchronization, as observed during sleep, has provided a good model for the study of interactions between synaptic plasticity and network state. A number of research groups have proposed different theories about how sleep affects brain activity and how it may contribute to the regulation of synaptic transmission. In this issue, the reviews by C. Cirelli and G. Tononi and by M. G. Frank present different viewpoints in this debate. The discussion generated by these reviews highlights the challenges that arise when attempting to connect findings from cellular, circuit, and system level approaches.

This special issue provides an up-to-date snapshot of a fundamental problem in neuroscience. All articles, while stating different opinions, converge in emphasizing the importance of homeostatic plasticity in maintaining stable performance of the nervous system. The richness of experimental and theoretical approaches now available will allow researchers to investigate neuron and network homeostasis in greater depth and detail and break new ground in the integration of findings from different levels of analysis. While the past years have been marked by several important discoveries in this field, there is still a long road ahead for connecting homeostatic mechanisms found at the cellular and network levels with actual maintenance of function at the systems level. 

